# The study on the application of solid-state method for synthesizing the polyaniline/noble metal (Au or Pt) hybrid materials

**DOI:** 10.1186/1556-276X-8-117

**Published:** 2013-03-02

**Authors:** Ruxangul Jamal, Feng Xu, Weiwei Shao, Tursun Abdiryim

**Affiliations:** 1 Key Laboratory of Petroleum and Gas Fine Chemicals, Educational Ministry of China, School of Chemistry and Chemical Engineering, Xinjiang University, Urumqi 830046, People’s Republic of China; 2 Key Laboratory of Functional Polymers, Xinjiang University, Urumqi 830046, People’s Republic of China; 3 Key Laboratory of Oil and Gas Fine Chemicals, Educational Ministry of China, College of Chemistry and Chemical Engineering, Xinjiang University, Urumqi 830046, People’s Republic of China

**Keywords:** Solid-state synthesis, Polyaniline, Noble metal, Hybrid materials, Sensor

## Abstract

The solid-state method was applied for synthesizing polyaniline (PANI)/noble metal hybrid materials with the presence of HAuCl_4_·4H_2_O or H_2_PtCl_6_·6H_2_O in the reaction medium. The structure, morphology, and electrochemical activity of the composites were characterized by Fourier transform infrared (FTIR) spectra, UV-visible (vis) absorption spectra, energy dispersive spectrum (EDS), X-ray powder diffraction (XRD), transmission electron microscopy (TEM), scanning electron microscopy (SEM), and cyclic voltammetry. The results from FTIR and UV-vis spectra showed that the oxidation degree and doping level of the PANI in composites can be influenced by HAuCl_4_·4H_2_O and H_2_PtCl_6_·6H_2_O. The EDS data demonstrated that the composites contain a certain amount of Au (or Pt) element. XRD analysis indicated the presence of crystalline-state Au particles in PANI matrix prepared from the presence of HAuCl_4_·4H_2_O and revealed that the H_2_PtCl_6_·6H_2_O cannot be converted into metal Pt. The TEM and SEM images implied that the Au particles did exist in the polymer matrix with the size of about 20 nm. The enzymeless H_2_O_2_ sensor constructed with PANI/Au composite from the presence of HAuCl_4_·4H_2_O showed a short response time (within 5 s) and displayed an excellent performance in wide linear range.

## Background

Noble metal nanoparticles such as Au and Pt nanoparticles have high catalytic activity, nontoxicity, and biocompatibility [1]. Conducting polymers are usually used as matrix to noble metal nanoparticles and then applied in biosensors [2,3], electrocatalysts [4], and supercapacitors [5], due to the synergy effect between polymer matrix and inorganic nanoparticles. Among various conducting polymers, polyaniline (PANI) has a potential use in a broad field because of its high environmental stability, low cost, relatively facile preparation, and reversible control of conductivity by charge-transfer doping and protonation [6]. The composite of PANI and Au (or Pt) nanoparticles, which have been intensively investigated, are also attractive materials as they combine the properties of large surface area, high conductivity, and excellent biocompatibility [7,8]. Up to now, PANI/Au (or Pt) hybrid material can be synthesized chemically or electrochemically. These methods have the advantages of easily controlling operating conditions. However, they have significant disadvantages such as the formation of toxic waste products and are not suitable for mass production. Solid-state synthesis is a mechanochemical reaction that occurs between powders in the solid state [9]. It is a new synthetic method to develop green chemistry with obvious advantages: reduced pollution, low costs, and simplicity in process and handling. Also, these factors are especially important in the industry.

H_2_O_2_ as a metabolic intermediate involved in many biological reactions plays an important role in the fields of chemistry, biology, clinical control, and environmental protection; therefore, its detection is of great importance [10]. To date, various techniques including spectrometry, titrimetry, chemiluminescence, and electrochemistry have been employed for determination [1,11,12]. Among the developed techniques, electrochemical methods have become one of the predominant analytical techniques due to their high sensitivity, low cost, and low power requirement [13]. Moreover, among the electrochemical methods, amperometric sensors have shown great potential for developing versatile analytical techniques for H_2_O_2_ determination [14]. The conducting polymer/metal composite amperometric enzyme electrodes as sensors have been paid particular attention due to their advantages of high sensitivity and specificity [14,15]. However, an efficient electron transfer between the active site of the enzyme and the electrode surface is not quite stable and depends on the enzyme type, temperature, and pH as a function of time [15]. Therefore, an alternative sensor called ‘enzymeless sensor’, which try to mimic natural enzymes with the same effectiveness and selectivity, has been widely studied [16,17].

Herein, we report the exploration of synthesizing the polyaniline/noble metal hybrid materials by solid-state synthesis method at room temperature. The structure, morphology, and components of composites were characterized by Fourier transform infrared (FTIR), UV-visible (vis), X-ray powder diffraction (XRD), energy dispersed spectrum (EDS), scanning electron microscopy (SEM), and transmission electron microscopy (TEM) methods. Furthermore, the composite from the existence of HAuCl_4_·4H_2_O in the reaction medium was selected for designing an enzymeless sensor on a glassy carbon electrode (GCE) for H_2_O_2_ detection.

## Methods

Aniline and ammonium peroxydisulfate were obtained from Xi’an Chemical Reagent Company (Xi’an, China). Chloroauric acid hydrated (HAuCl_4_·4H_2_O), chloroplatinic acid hydrated (H_2_PtCl_6_·6H_2_O), and *p*-toluenesulfonic acid (*p*-TSA) were purchased from Shanghai Aladdin Reagent Company (Shanghai, China). H_2_O_2_ (30 wt.%) was obtained from Tianjin Chemical Reagent Company (Tianjin, China). Nafion, a 5-wt.% solution in a mixture of lower aliphatic alcohols and 20% water, was obtained from Sigma-Aldrich (St. Louis, MO, USA). Before use, it was diluted with 0.5 wt.% isopropanol. All the reagents were of analytical grade, aniline was purified by distillation under reduced pressure and stored in a refrigerator, and all other chemicals and solvents were used as received without further purification. Phosphate buffer saline (PBS; 0.1 M) was prepared by mixing stock solutions of NaH_2_PO_4_ and Na_2_HPO_4_.

**Figure 1:**
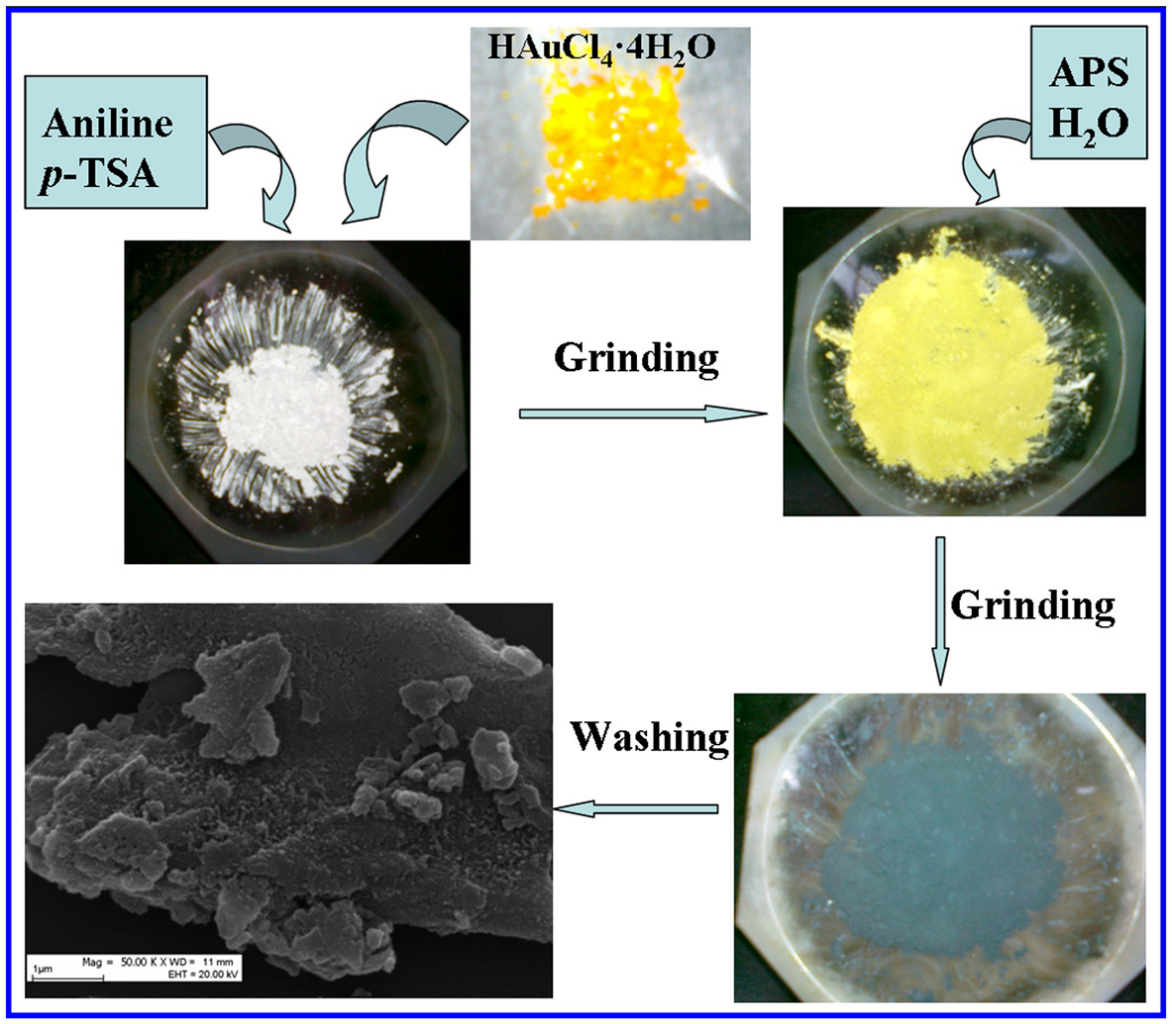
**Schematic of solid-state method synthesis of PANI(HAuCl**_**4**_**·4H**_**2**_**O) hybrid material.**

A typical solid-state synthesis process for the composites was as follows (as shown in Figure 1): 1 mL aniline was added quickly to the mortars containing *p*-TSA (1.9 g). After grinding for about 10 min, 0.1 g yellowish-red crystalloid HAuCl_4_·4H_2_O (10.0 wt.% of the aniline monomer) and 1 mL H_2_O were added and ground homogeneously for 5 min, then 2.28 g was added, and the mixture was further ground for 30 min. The obtained powder was washed with ethanol and distilled water until the filtrate was colorless, and then the powder was dried under vacuum at 60°C for 48 h. The obtained hybrid materials were denoted as PANI(HAuCl_4_·4H_2_O), which indicated that the composite was prepared from the reaction system with the existence of HAuCl_4_·4H_2_O. In a similar manner, we also prepared the composite with the presence of the same amount of H_2_PtCl_6_·6H_2_O (10.0 wt.% of the aniline monomer) in the reaction medium, and the composite was denoted as PANI(H_2_PtCl_6_·6H_2_O), which indicated that the composite was prepared from the reaction system with the existence of H_2_PtCl_6_·6H_2_O. Pure PANI had also been prepared using the above-mentioned procedure. The yield of samples were 0.56 and 0.47 g for the PANI(HAuCl_4_·4H_2_O) and PANI(H_2_PtCl_6_·6H_2_O), respectively.

The FTIR spectra of the composites were obtained using a Bruker Equinox-55 Fourier transform infrared spectrometer (Bruker, Billerica, MA, USA) (frequency range 4,000 to 500 cm^−1^). The UV-vis spectra of the samples were recorded on a UV-vis spectrophotometer (UV4802, Unico, Dayton, NJ, USA). XRD patterns have been obtained using a Bruker AXS D8 diffractometer with monochromatic Cu Kα radiation source (*λ* = 0.15418 nm), the scan range (2*θ*) was 5° to 70°. SEM measurements were performed on a Leo 1430VP microscope (Zeiss, Oberkochen, Germany) with Oxford Instruments (Abingdon, Oxfordshire, UK). EDS experiments were carried out with a pellet which was pressed at 200 MPa and then adhered to copper platens.

A three-electrode system was employed to study the electrochemical performances of composites. Pt electrode was used as a counter electrode and saturated calomel electrode as a reference electrode. PANI(HAuCl_4_·4H_2_O)-modified GCE (diameter = 3 mm) was used as a working electrode. The working electrode was fabricated by placing a 5-μL dispersion (30 mg/L) on a bare GCE surface and air-dried for 10 min. All the experiments were carried out at ambient temperature and air atmosphere.

## Results and discussion

**Figure 2:**
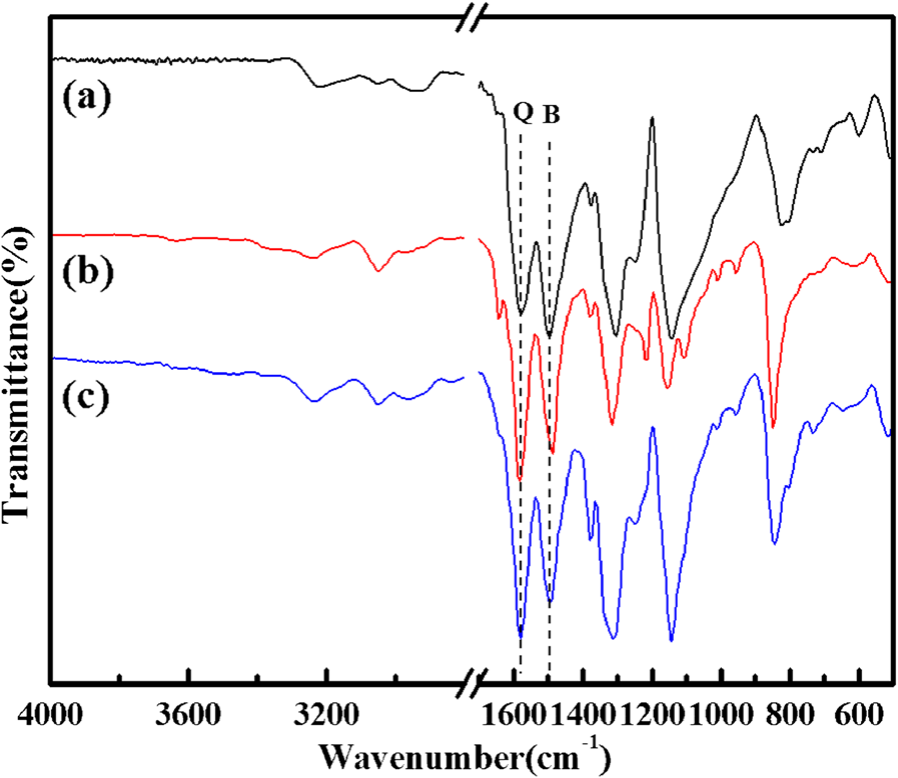
**FTIR spectra.** Curves (**a**) PANI, (**b**) PANI(HAuCl_4_·4H_2_O), and (**c**) PANI(H_2_PtCl_6_·6H_2_O).

Figure 2 shows the FTIR spectra of the pure PANI, PANI(HAuCl_4_·4H_2_O), and PANI(H_2_PtCl_6_·6H_2_O). As shown in Figure 2, the FTIR spectra of PANI(HAuCl_4_·4H_2_O) and PANI(H_2_PtCl_6_·6H_2_O) are almost identical to that of PANI. The band at approximately 3,235 cm^−1^ is attributable to the N-H stretching vibration [18], while the two bands appearing at approximately 1,580 and 1,493 cm^−1^ are associated to the stretching vibration of nitrogen quinoid (Q) and benzenoid (B) rings, respectively [19]. The band at approximately 1,315 cm^−1^ can be assigned to the C-N mode [20], while the band at approximately 1,146 cm^−1^ is the characteristic band of the stretching vibration of quinoid, and the band appearing at approximately 820 cm^−1^ is attributed to an aromatic C-H out-of-plane bending vibration [19].

Generally, the Q/B (*I*_~1,580 cm_^−1^/*I*_~1493 cm_^−1^) value indicates the oxidation degree of PANI [21]. A comparison indicates that the composites exhibit a higher intensity ratio of Q to B ring modes than pure PANI, suggesting that there are more quinoid units in the composites than pure PANI. This result can be attributed to the adding of HAuCl_4_ and H_2_PtCl_6_, which can serve not only as the resource of metal particles, but also as strong oxidants, which can enhance the oxidation degree of the PANI in composites [22,23].

**Figure 3:**
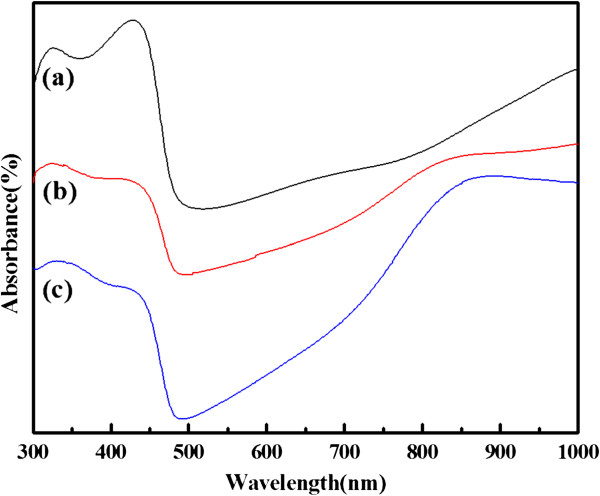
**UV-vis spectra.** Curves (**a**) PANI, (**b**) PANI(HAuCl_4_·4H_2_O), and (**c**) PANI(H_2_PtCl_6_·6H_2_O).

Figure 3 represents the UV-vis absorption spectra of PANI, PANI(HAuCl_4_·4H_2_O), and PANI(H_2_PtCl_6_·6H_2_O) in *m*-cresol solution. The characteristic peaks of PANI and composites at approximately 320 to 330 nm, approximately 430 to 445 nm, and 820 to 870 nm are attributed to *π*-π*, polaron-π*, and *π*-polaron transitions, respectively [18]. Feng et al. reported that pure Au nanoparticles usually show an absorption peak at approximately 510 nm as a result of the surface plasmon resonance [24], whereas Pt nanoparticles usually have no absorption peak at 300 to 1,000 nm [25,26]. However, in this case, the surface plasmon resonance bands of Au nanoparticles are not observed, which may be caused by the changing of their surrounding environment [7]. However, the absorption peaks of *π*-polaron change significantly, and the intensity ratio (A_820–870_/A_320–330_) of the composites is higher than PANI, indicating that the doping level of the PANI in composites is higher than that of pure PANI [27]. Therefore, the results from the UV-vis absorption spectra imply that the HAuCl_4_ or H_2_PtCl_6_ have certain effects on the polymer chains.

**Figure 4:**
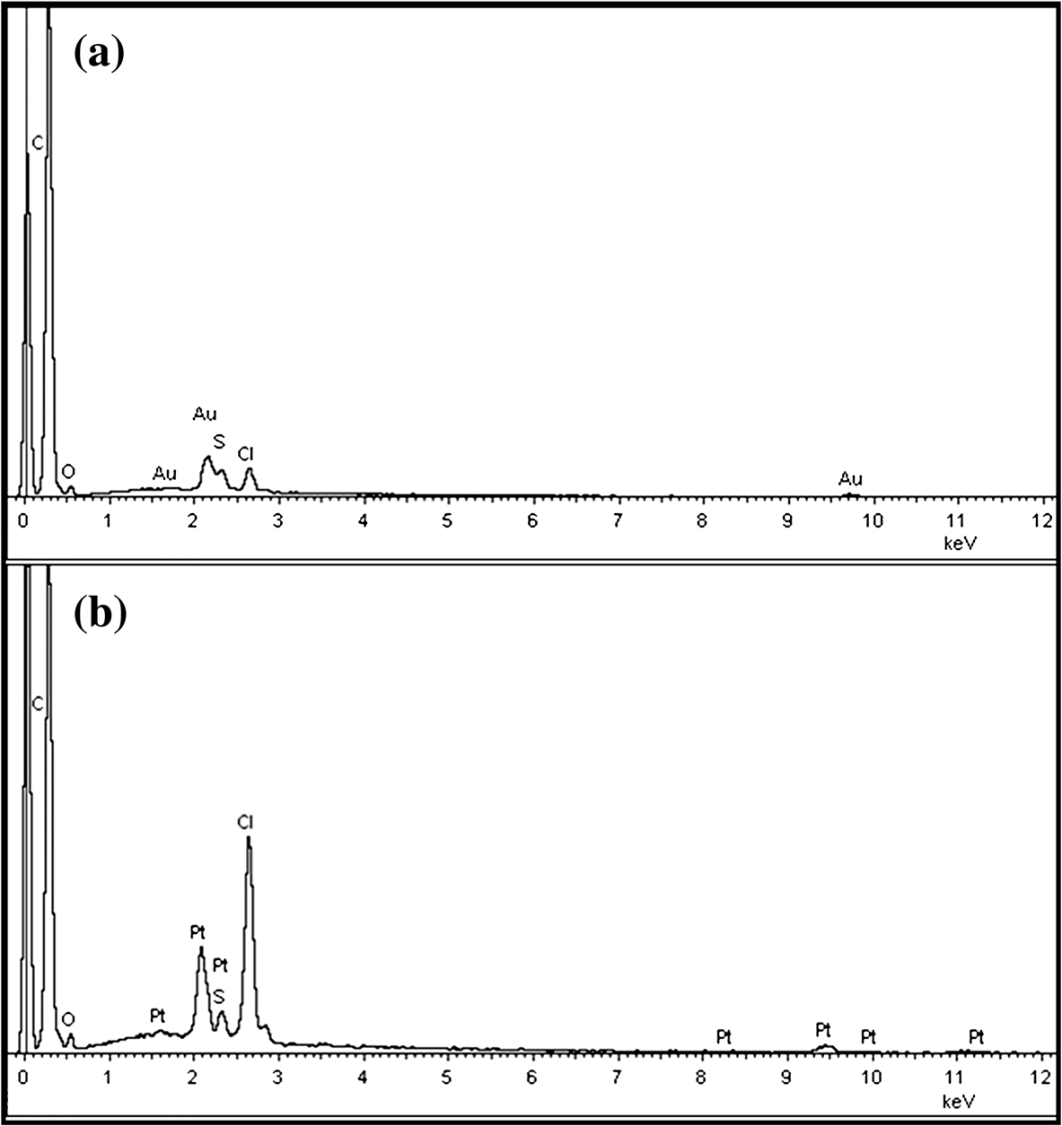
**EDS spectra of composites.** (**a**) PANI(HAuCl_4_·4H_2_O) and (**b**) PANI(H_2_PtCl_6_·6H_2_O).

**Figure 5:**
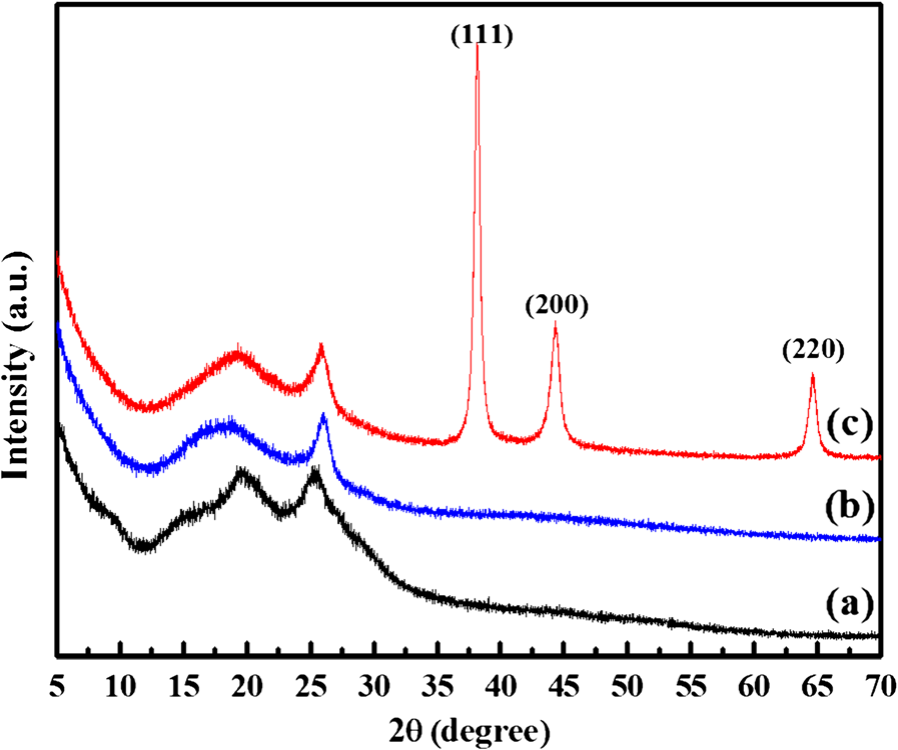
**XRD patterns.** Curves (**a**) PANI, (**b**) PANI(H_2_PtCl_6_·6H_2_O), and (**c**) PANI(HAuCl_4_·4H_2_O).

**Figure 6:**
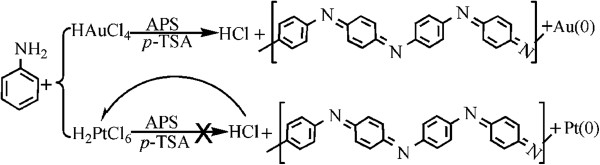
**Schematic of a possible mechanism for the formation of hybrid materials of PANI(HAuCl**_**4**_**·4H**_**2**_**O) and PANI(H**_**2**_**PtCl**_**6**_**·6H**_**2**_**O).**

Figure 4 is the EDS of the composites. It can be concluded from Figure 4 that the Au and Pt elements do exist in the polymer matrix, and the weight percentages are 7.65 and 6.07 for Au and Pt elements, respectively. Figure 5 shows the XRD patterns of PANI, PANI(HAuCl_4_·4H_2_O), and PANI(H_2_PtCl_6_·6H_2_O). As indicated in Figure 5, the PANI exhibits two peaks at 2*θ* approximately 20° and approximately 26°, which are ascribed to the periodicity parallel and perpendicular to the polymer chains, respectively [28]. In the case of PANI(HAuCl_4_·4H_2_O), the strong peaks appeared at 2*θ* values of 38°, 44°, and 64.5° which can be assigned to Bragg's reflections from the (111), (200), and (220) planes of metal Au [3]. These Bragg's reflections are in good agreement with the data (JCPDS-ICCD, 870720), which can further prove the existence of Au nanoparticles in the PANI(HAuCl_4_·4H_2_O). However, there is no characteristic Bragg's reflection for metal Pt in the case of PANI(H_2_PtCl_6_·6H_2_O), which is a similar phenomenon to that of Pt nanoparticles deposited on carbon nanotubes using PANI as dispersant and stabilizer [29]. Combined with the results from EDS analysis, it can be concluded that the Pt element may exist in the form of [PtCl_6_]^2−^, [PtCl_5_(H_2_O)]^−^, and [PtCl_4_(H_2_O)_2_] in the polymer matrix because the deprotonation reaction of the aqua ligands of H_2_PtCl_6_ are fully suppressed by the high concentration of protons in the reaction system by solid-state method [30]. However, these interesting results indicate the potential application of the solid-state method for polymer complex such as PANI-type conducting polymers Pt(IV) complexes. The general reactions for the reduction of HAuCl_4_ and H_2_PtCl_6_ by PANI in this reaction are illustrated in Figure 6[7,31].

**Figure 7:**
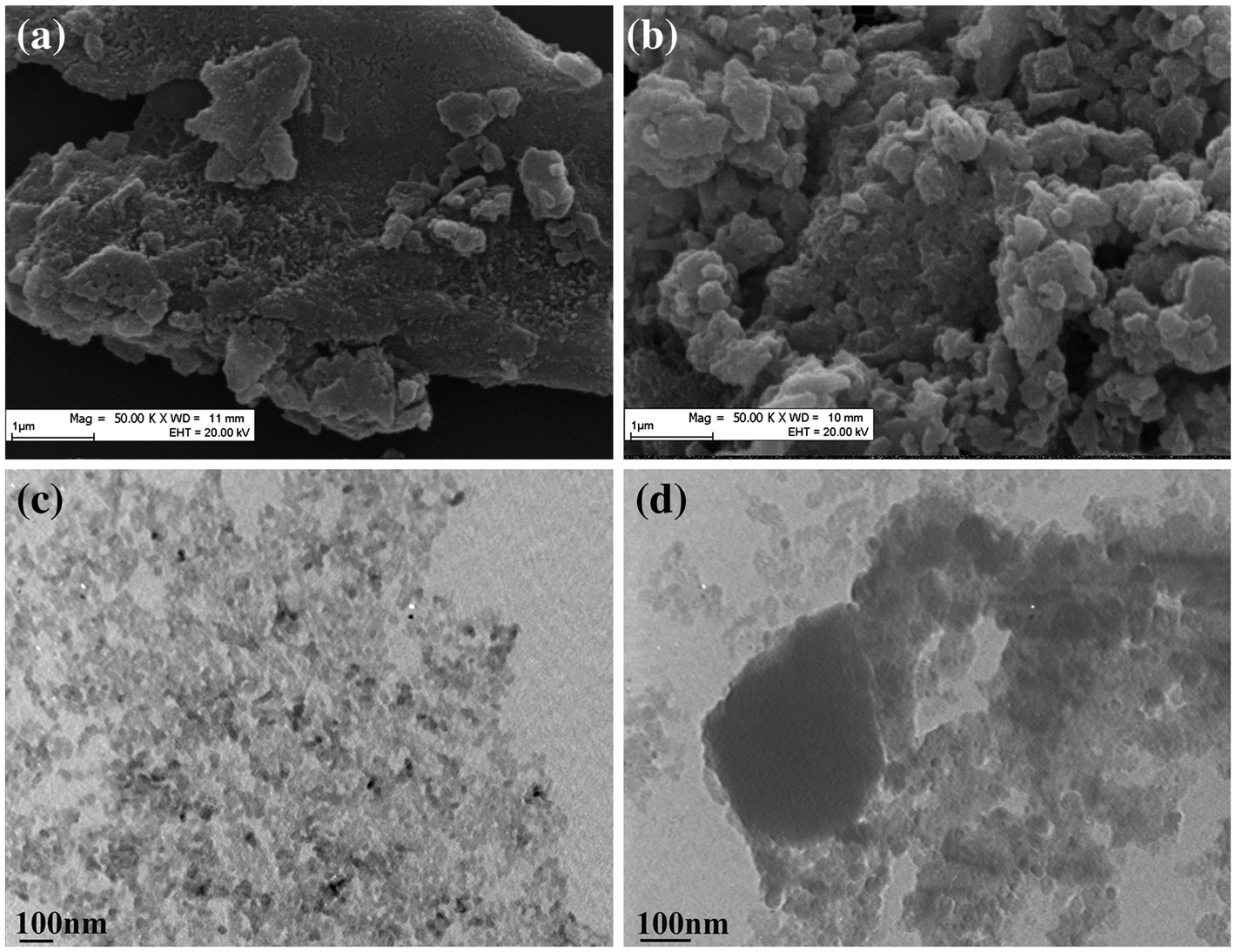
**TEM and SEM images of PANI(HAuCl**_**4**_**·4H**_**2**_**O) and PANI(H**_**2**_**PtCl**_**6**_**·6H**_**2**_**O).** (**a**) SEM and (**c**) TEM images of PANI(HAuCl_4_·4H_2_O); (**b**) SEM and (**d**) TEM images of PANI(H_2_PtCl_6_·6H_2_O).

Figure 7 indicates the SEM and TEM images of the PANI(HAuCl_4_·4H_2_O) and PANI(H_2_PtCl_6_·6H_2_O). As shown in the SEM and TEM images, the size and shape of PANI particles are irregular. Some Au nanoparticles (the bright spots in Figure 7a) disperse better in the surface of the PANI matrix. However, based on the results of EDS analysis, it can be concluded that the total amount of Au nanoparticles (7.65 wt.%) is not very well consistent with the estimated value of 10 wt.% (assuming all the Au salt is converted to Au(0)). If one considers the conversion rate of Au salt to Au nanoparticles in this solid-state reaction, the value of conversion rate is about 89.6% (Conversion rate = (Yield of sample) × (Elemental percentage of Au)/(Au in 100 mg HAuCl_4_·4H_2_O)). In addition, it is evident from Figure 7c that the size of the Au nanoparticles (the sand-like dark spots in Figure 7c) is about 20 nm. However, in the case of PANI(H_2_PtCl_6_·6H_2_O), there are not any Pt metal particles found in either SEM or TEM images. This phenomenon is consistent with the results of XRD patterns.

**Figure 8:**
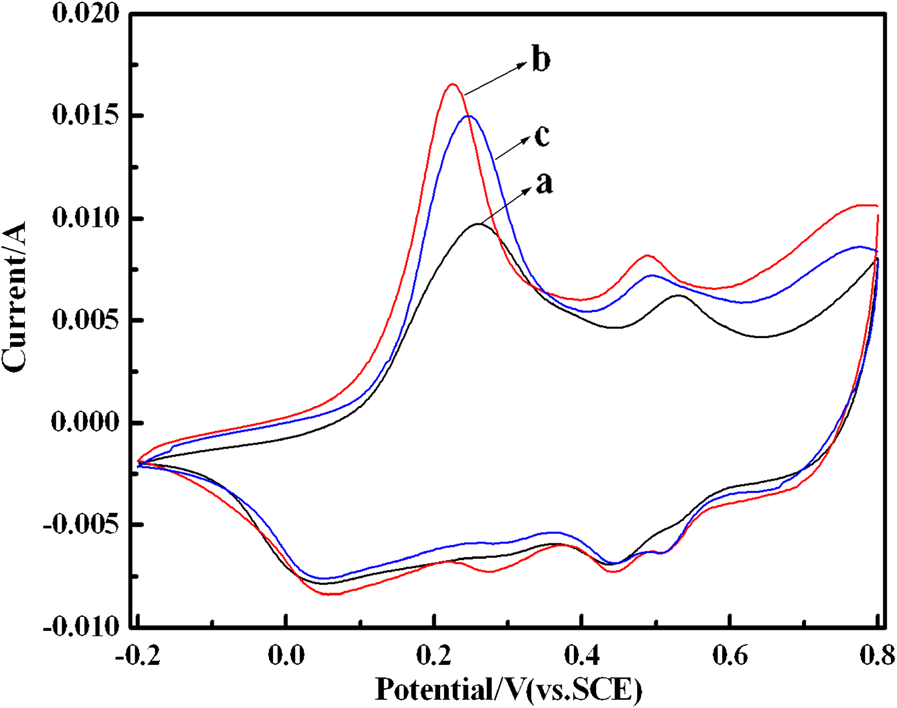
**CV curves of PANI (a), PANI(HAuCl**_**4**_·**4H**_**2**_**O) (b), and PANI(H**_**2**_**PtCl**_**6**_**·6H**_**2**_**O) (c) in 1 M H**_**2**_**SO**_**4 **_**electrolyte.** Scan rate is 3 mV s^−1^. Mass of the active material is 3 mg, and graphite current collector was used (area 1 cm^2^) as the working electrode.

Figure 8 shows the cyclic voltammetry (CV) curves of PANI, PANI(HAuCl_4_·4H_2_O), and PANI(H_2_PtCl_6_·6H_2_O) electrodes measured from −0.2 to 0.8 V in 1 M H_2_SO_4_ electrolyte. Overall, the redox peaks of composites are similar to the pure PANI, indicating that the HAuCl_4_ and H_2_PtCl_6_ cannot affect the formation of PANI in composites. However, a comparison demonstrates that the oxidation peak currents of composites are higher than those of pure PANI and shift negatively to a lower potential range than those of pure PANI. This phenomenon can be associated to the higher oxidation degree and doping level of the PANI in composites than that of pure PANI, which can improve the electrochemical activity of composites. Moreover, the oxidation potential of PANI(HAuCl_4_·4H_2_O) shifts to lower potential than those of others, which may be a result of the Au nanoparticles possibly enhancing the flow ability of electron in the polymer chain [2].

**Figure 9:**
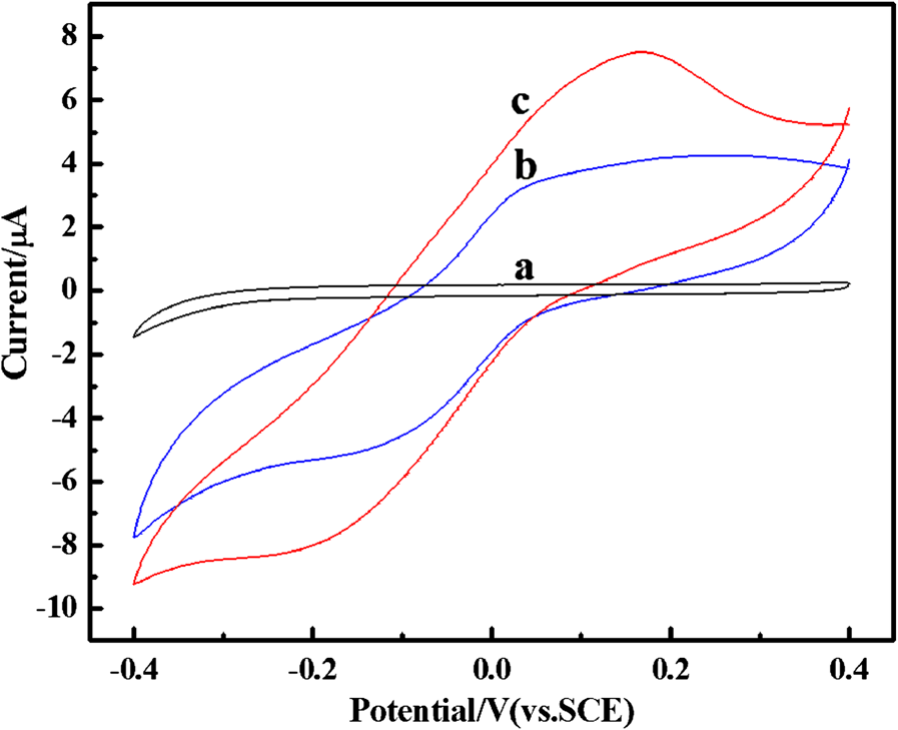
**CV curves of bare GCE and PANI(HAuCl**_**4**_·**4H**_**2**_**O)/GCE.** GCE (curve a) and PANI(HAuCl_4_·4H_2_O)/GCE in 0.1 M PBS at pH 6.8 without (curve b) and with (curve c)10 mM H_2_O_2_. Scan rate is 50 mV s^−1^.

As the XRD patterns of PANI(H_2_PtCl_6_·6H_2_O) did not show any characteristic Bragg's reflection for metal Pt, the PANI(HAuCl_4_·4H_2_O) was selected as a type of catalyzing electrode material, and an enzymeless H_2_O_2_ sensor was assembled by the dripping of the dispersion of PANI(HAuCl_4_·4H_2_O) on a GCE surface. Figure 9 shows the electrocatalytic responses of bare GCE and PANI(HAuCl_4_·4H_2_O)/GCE in 0.1 M PBS at pH 6.8 with and without 10 mM H_2_O_2_. It is clear that that there is no evident redox peak observed on a bare GCE which is due to the lack of substance with electrochemical activity. On the contrary, the PANI(HAuCl_4_·4H_2_O)/GCE shows a pair of reduction (5 μA at −0.15 V) and oxidation (3 μA at 0.15 V) peak currents. It is common that PANI showed one pair of peaks in neutral pH environment [32]. It is also important to note that both the reduction and oxidation current for PANI(HAuCl_4_·4H_2_O)/GCE increased after addition of H_2_O_2_. These observations indicate that PANI(HAuCl_4_·4H_2_O)/GCE can act as catalysts for both the reduction and oxidation of H_2_O_2_.

**Figure 10:**
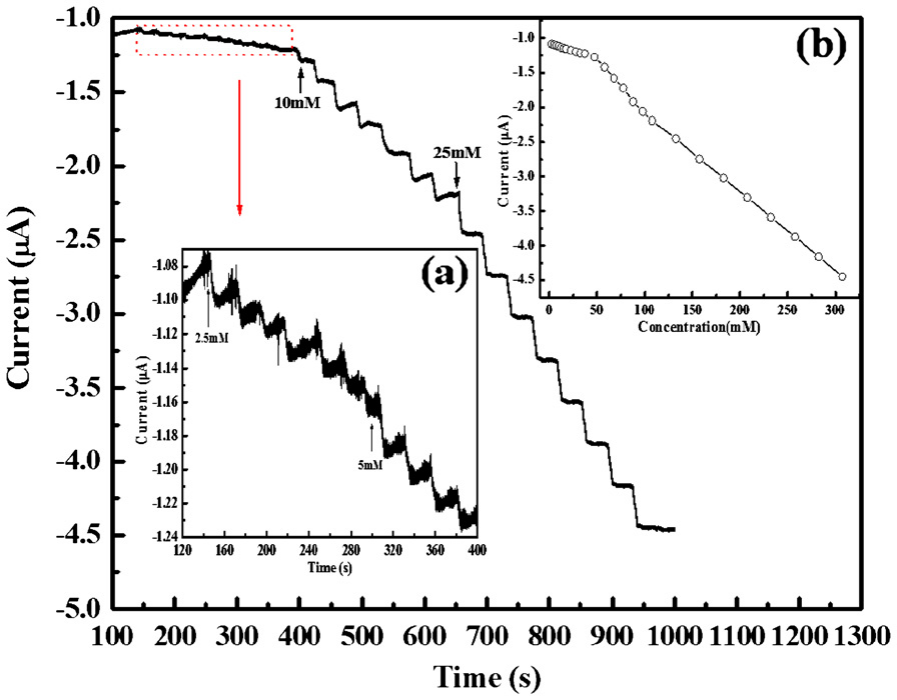
**Amperometric response of the enzymeless sensor to H**_**2**_**O**_**2**_**.** The applied potential is −0.2 V in 0.1 M PBS at pH 6.8. Inset (**a**) shows a magnification of the 120 to 400 s additions of H_2_O_2_, and inset (**b**) shows the steady-state current vs. H_2_O_2_ concentration.

The amperometric response of the enzymeless H_2_O_2_ amperometric sensor was investigated by successively adding H_2_O_2_ to a continuous stirring of 20 mL 0.1 M PBS at pH 6.8. Figure 10 demonstrates the typical current-time curve of the enzymeless sensor. As can be seen in Figure 10, a sharp increase in the current is observed in negative within a response time of less than 5 s after each addition of H_2_O_2_ direction, which is lower than the amperometric response(<2 s) of enzyme biosensor based on *in situ* electrosynthesized PANI/Au core-shell nanocomposite [14]. However, the linear regression equation was *i* = −0.9256 − 0.0057[H_2_O_2_] (mM), with a correlation coefficient of 0.997 (inset b in Figure 10). This reveals that this non-enzymatic sensor shows similar performance in terms of wide linear range compared with enzyme-based biosensor [14].

## Conclusions

In this paper, the synthesis of the polyaniline/noble metal hybrid materials by solid-state method in the presence of HAuCl_4_·4H_2_O or H_2_PtCl_6_·6H_2_O in the reaction system was investigated. These composites were characterized by FTIR, UV-vis, X-ray, TEM, SEM, and EDS as well as by the electrochemical measurements. The results showed that the strong oxidation ability of HAuCl_4_·4H_2_O and H_2_PtCl_6_·6H_2_O was a main factor in increasing the oxidation degree and doping level of the PANI in composites. Furthermore, the results also indicated that the HAuCl_4_·4H_2_O can be converted into Au nanoparticles, while that of the H_2_PtCl_6_·6H_2_O cannot be converted into metal Pt, suggesting the formation of [PtCl_6_]^2−^, [PtCl_5_(H_2_O)]^−^, and [PtCl_4_(H_2_O)_2_] in the polymer matrix. Compared with the existing methods, the method demonstrated here was facile but effective and could be readily used for a large-scale preparation of the PANI/Au. However, the PANI/Pt was not successfully synthesized by this solid-sate method which may be a result of the fully suppressed deprotonation reaction of aqua ligands of H_2_PtCl_6_ by the high concentration of protons in the reaction system. These interesting results indicated the potential application of the solid-state method for polymer complex such as PANI-type conducting polymer Pt(IV) complexes. Furthermore, the electrochemical measurements indicated that the obtained PANI/Au displayed a fast response to H_2_O_2_ and excellent performance in wide linear range. The sensor could catalyze the oxidation and reduction of H_2_O_2_ at the same time, and it exhibited a fast amperometric response (about 5 s) to the reduction of H_2_O_2_ in a wide linear range.

## Competing interests

The authors declare that they have no competing interests.

## Authors’ contributions

RJ conceived the study, carried out data analysis, and drafted the manuscript. FX carried out the sample preparation and the experimental measure. WS participated in the study of material structures and the data analysis. TA coordinated the research and revised and finalized the manuscript. All authors read and approved the final version of the manuscript.
